# Distinct histone H3 modification profiles correlate with aggressive characteristics of salivary gland neoplasms

**DOI:** 10.1038/s41598-022-19174-9

**Published:** 2022-09-05

**Authors:** Aroonwan Lam-Ubol, Ekarat Phattarataratip

**Affiliations:** 1 Department of Oral Surgery and Oral Medicine, Faculty of Dentistry, Srinakharinwirot University, 114 Sukhumvit 23 Wattana, Bangkok, 10110 Thailand; 2 Department of Oral Pathology, Faculty of Dentistry, Chulalongkorn University, Henri-Dunant Road, Pathumwan, Bangkok, 10330 Thailand

**Keywords:** Head and neck cancer, Oral cancer

## Abstract

Post-translational modification of histones is the crucial event that affect many tumor-specific traits. A diverse type of histone modifications had been reported in different cancers with prognostic implications. This study aimed to examine the degree of histone H3 modifications in salivary gland neoplasms and their associations with tumor pathologic characteristics and proliferative activity. The expression of H3K9Ac, H3K18Ac, H3K9Me3 and Ki-67 in 70 specimens of salivary gland neoplasms, consisting of 30 mucoepidermoid carcinoma (MEC), 20 adenoid cystic carcinoma (ACC) and 20 pleomorphic adenoma (PA), were investigated immunohistochemically. The immunohistochemical scoring of 3 histone modification types and Ki-67 labeling index were determined. Overall, MEC demonstrated elevated H3K9Ac level compared with benign PA. Increased H3K9Me3 in MEC was positively correlated with small nest invasion at tumor front, advanced pathologic grade, and elevated proliferative index. In addition, the significant upregulation of all 3 types of histone H3 modification was noted in solid subtype of ACC and associated with increased cell proliferation. This study indicates that salivary gland neoplasms differentially acquire distinct patterns of histone H3 modification, which impact prognostically relevant cancer phenotypes. The hyperacetylation and methylation of histone H3 could be underpinning the prognostically worsen solid type of ACC, and the trimethylation of H3K9 may be involved in aggressive characteristics of MEC.

## Introduction

Tumorigenesis is a multistep process, shaped by both the genetic and epigenetic input. The genetic mutations involving the activation of oncogenes and inactivation of tumor suppressor genes have long been considered the central molecular pathogenesis of cancer. Increasing evidence has indicated the critical role of disrupted epigenetic phenomena, leading to the epigenomic instability that triggers the tumor formation^1^.

Post-translational modification primarily at the N-terminal amino acid tails of the core H3 and H4 is the one of the vital events that can alter the structure and function of genome and impact the transcription of various target genes, responsible for cancer-specific characteristics. Differential histone modifications along with their prognostic implication had been reported in various cancers^2–14^. Acetylation and methylation involving lysine residue have been the most intensely studied histone modifications. Histone acetylation is known to diminish histone-DNA affinity and thereby releases chromatin configuration to enhance gene transcription. In contrast, histone methylation can affect chromatin structures differently, depending on the site and type of amino acid residues methylated^15^. The hypermethylation of histone H3 lysine 9 (H3K9) is particularly associated with the compact chromatin formation and transcriptional repression of target genes^16^. Aberrant expression of histone modifications as well as responsible enzymes, such as histone methyltransferases (HMT), histone acetyltransferase (HAT), and histone deacetylase (HDAC) were reported in several types of cancer. Suv39H1 and SETDB1, the two main HMTs that catalyzed heterochromatic H3K9Me3 deposition^17^, were shown to be overexpressed in selected cancers^18,19^. In addition, the upregulation of a number of HDACs were reported and inhibitors of histone modification enzymes had been developed for anti-cancer use with variable success^18^.

The effect of histone modification appears to be tumor type-specific, suggesting that these changes may govern distinct underlying mechanisms in different neoplasms. In addition, conflicting results have been reported within the same tumor type. In non-small cell lung cancers, the decreased acetylated histone H3 lysine 9 (H3K9Ac) and trimethylated histone H3 lysine 9 (H3K9me3) were associated with clinically recurrent lesions^13^. However, in patients with stage I lung adenocarcinoma, the lower level of H3K9Ac appeared to be associated with better disease-free and overall survival^2^.

In breast cancers, lower levels of lysine acetylation and methylation were predominantly seen in those with poor prognosis, including the HER-2-positive and basal cell carcinomas^4^. Leszinski et al*.* reported that the level of H3K9Me3 was upregulated in breast cancers, but down-regulated in colorectal cancers^7^. However, in the invasive portion of colorectal cancers, the H3K9Me3 was shown to be upregulated, and this finding was correlated with enhanced lymph node metastasis^20^. In gastric adenocarcinoma, high levels of H3K9Me3 was correlated with the increased cancer stage, lymphovascular invasion, recurrence and poor survival^10^.

The acetylated histone H3 lysine 18 (H3K18Ac) was increased in primary and metastatic prostate cancer specimens, compared with benign lesions^3^. Bianco-Miotto et al*.* reported the association between high H3K18Ac and increased prostate cancer relapse^21^. Conversely, in low-grade prostate cancers, the combination of lower H3K18Ac and acetylated histone H4 lysine 12 (H4K12Ac) levels correlated with the increased recurrence^12^. Low H3K18Ac expression was correlated with better prognosis of esophageal squamous cell carcinoma patients^14^, but could predict renal cell carcinoma progression^9^. In pancreatic cancer, Manuyakorn et al*.* showed that low H3K18Ac was associated with poor survival in stage I and II pancreatic adenocarcinomas^8^, whereas a more recent study by Juliano et al*.* reported the association between high H3K18Ac and advanced clinical staging as well as poor survival^6^.

Salivary gland neoplasms, representing 5% of head and neck neoplasms, constitute a unique group of benign and malignant entities with diverse tumor types, cells of origin, molecular genetic background, and clinical behavior. Most epigenetic studies involving salivary gland neoplasms have focused on the DNA methylation with a handful of studies examining the roles of histone modification in their progression. Wagner et al*.* reported that salivary gland malignancies were frequently hypoacetylated at H3K9, compared with benign tumors, and this was correlated with the increased Ki67 index^22^. In adenoid cystic carcinoma (ACC), the increased H3K9Me3 was noted in the solid subtype and was an independent predictor of poor disease-free survival^23^. Moreover, aberrant expression of a few HMTs and HDACs were reported in salivary gland neoplasms^24,25^. These data suggested that distinct histone modifications may play essential roles in salivary gland tumor progression. Additionally, several targeted therapies directed against specific types of histone modification are currently available and may provide promising prospect for patients with neoplasms of appropriate epigenetic signature. Therefore, the objectives of this study were to explore the pattern of global histone H3 modifications (H3K9Ac, H3K18Ac and H3K9Me3) in benign and malignant salivary gland neoplasms and analyze their associations with proliferative activity and prognostically-relevant pathologic characteristics.

## Materials and methods

### Tissue samples

The tissue samples were retrieved from the Department of Oral Pathology archival cases from 2000 to 2021. The inclusion criteria were that patients diagnosed with mucoepidermoid carcinomas (MEC), ACC and pleomorphic adenomas (PA) with complete pertinent clinical records and adequate paraffin-embedded tissue specimens. All microscopic slides were reviewed based on the 2017 World Health Organization Classification of Head and Neck tumor criteria. Microscopic grading of MEC was based on the Brandwein et al. histopathological criteria^26^. Prognostically relevant histopathologic characteristics, namely < 25% intracystic components, perineural, vascular or bone invasions, necrosis, mitotic index > 4/10 high power fields (HPFs), anaplasia and tumor front invasion in small nests were recorded. Histopathologically, ACCs were evaluated and categorized into 3 groups; the cribriform, tubular or solid subtypes. Cases with more than 30% of tumor showing solid sheet arrangement were categorized as the solid subtype. The remaining cases were classified into either cribriform or tubular subtypes, based on the predominant microscopic pattern. The study was approved by the Human Research Ethics Committee (HREC-DCU 2019-082).

### Immunohistochemical staining methods

The 2-µm tissue sections were deparaffinized. The antigen retrieval was performed by incubating slides with 1 mM Citrate buffer (pH 6.0) for 5 min in a pressure cooker. Slides were then incubated with 3% hydrogen peroxide for 10 min, followed by 30-min incubation with bovine serum albumin. The primary antibody was added and incubated at 4 °C overnight. The antibodies used were rabbit polyclonal anti-histone H3 (acetyl K18) (clone ab1191; dilution 1:400), anti-histone H3 (acetyl K9) (clone ab10812; dilution 1:1000), anti-histone H3 (tri-methyl K9) (clone ab8898; dilution 1:400) (Abcam, Cambridge, UK) and mouse monoclonal anti-Ki-67 (clone MIB-1, 1:100 dilution) (Agilent Dako, Glostrup, Denmark) antibodies. This was followed by a 60-min incubation with goat anti-rabbit secondary antibody (clone P0448, Agilent Dako, Santa Clara, CA) for 3 types of histone modification tested, or the EnVision + System-HRP Labelled Polymer Anti-mouse (K4001, Agilent Dako, Glostrup, Denmark) for Ki-67 staining. Slides were then incubated for 1 min with diaminobenzidine (Liquid DAB + Substrate Chromogen System, K3468, Agilent Dako, Santa Clara, CA) and counterstained with Mayer’s Haematoxylin. Slides were rinsed with phosphate-buffered saline between each step. Oral squamous cell carcinoma tissues were used as positive controls. Immunostainings of adjacent normal salivary gland tissues were examined for comparison purpose. Negative controls are prepared using isotype-matched control antibodies.

### Immunohistochemical scoring

The immunohistochemical staining was evaluated and agreed upon by two experienced pathologists who were blinded from the patient clinical data. For scoring histone modification levels, both the intensity and distribution of positive nuclear staining in tumor cells were examined. The staining intensity of each case was categorized into one of the following levels: 0 = no positive staining; 1 = mild intensity; 2 = moderate intensity and 3 = strong intensity. The percentage of positive tumor cells of each case was recorded. The H-score was calculated as previously described by multiplying the staining intensity level and the percentage of positive tumor cells, to represent the expression level of individual cases (0–300)^27^. Levels of H-score were categorized as low (H-score ≤ 150) and high (H-score > 150) expression.

Tumor cells with Ki-67 nuclear staining were counted as described previously^28^. Briefly, the photomicrographs of 3 fields with highest positivity in each slide were taken at 400× by Canon EOS 600D EOS Digital SLR Camera. Oral and Maxillofacial pathologist counted at least 500 tumor cells from each case. Ki-67 labeling index (LI), determined by the ratio between positive cells and total cell count, was recorded. The Ki-67 expression was categorized into 2 groups of low (LI ≤ 1.25) and high (LI > 1.25) proliferative activity.

### Statistical analysis

The results were statistically analyzed using the IBM SPSS Statistics version 21 (IBM Corporation, NY). The continuous variables were expressed as means ± standard deviation (SD). Categorical analyses of the Ki-67 LI, the clinical-pathologic characteristics and the expression levels of H3K9Ac, H3K18Ac and H3K9Me3 were performed using non-parametric Mann–Whitney U or Kruskal–Wallis test as appropriate. Bonferroni correction for multiple comparison was applied to adjust the significant values. Spearman’s rank correlation was used to assess the associations among the levels of modified histone H3, defined by H-scores in each neoplasm. A *p*-value less than 0.05 was considered statistically significant. Receiver operating characteristic (ROC) curves and area under curve (AUC) were generated by GraphPad Prism version 9.4.0.

### Ethics approval and consent to participate

All procedures involving human participants in this study were in accordance with the ethical standards of the institutional research committee and with the 1964 Helsinki declaration and its later amendments or comparable ethical standards. The Human Research Ethics Committee of Faculty of Dentistry, Chulalongkorn University approved the study protocol (HREC-DCU 2019-082) and waived the need of informed consent for this study.

## Results

### Demographic data of study population

A total of 70 specimens of salivary gland neoplasms, i.e., 30 MECs, 20 ACCs and 20 PAs, were included in this study. The majority of specimens were from minor salivary glands with hard palate being the most common site. Regarding the histopathologic grading, MEC was composed of 14, 7 and 9 cases of low-, intermediate- and high-grades, respectively. ACC comprised 6 solid and 14 cribriform/tubular subtypes. Clinico-pathologic characteristics features of the study population are summarized in Table 1.

**Table 1:** Demographic data of the study population.

Patient characteristics	MEC (N = 30)	ACC (N = 20)	PA (N = 20)
**Age (years)**			
Mean	45.7 ± 19.5	43.45 ± 15.6	43.6 ± 15.2
Range	16–82	16–73	21–74
**Sex (N (%))**			
Male	12 (40)	10 (50)	8 (40)
Female	18 (60)	10 (50)	12 (60)
**Sites (N (%))**			
Hard palate	17 (56.7)	13 (65)	14 (70)
Soft palate	3 (10)	2 (10)	0
Alveolar mucosa	5 (16.7)	1 (5)	0
Retromolar mucosa	3 (10)	1 (5)	0
Buccal mucosa	1 (3.3)	2 (10)	2 (10)
Floor of mouth	0	1 (5)	0
Upper lip	0	0	3 (15)
Submandibular gland	1 (3.3)	0	1 (5)
**Clinical size (cm)**			
Mean	3 ± 1.45	3.2 ± 1.2	3.15 ± 2.3
Range	1-6	1.5–5	1-9

### The proliferative activity of salivary gland neoplasms

**Figure 1:**
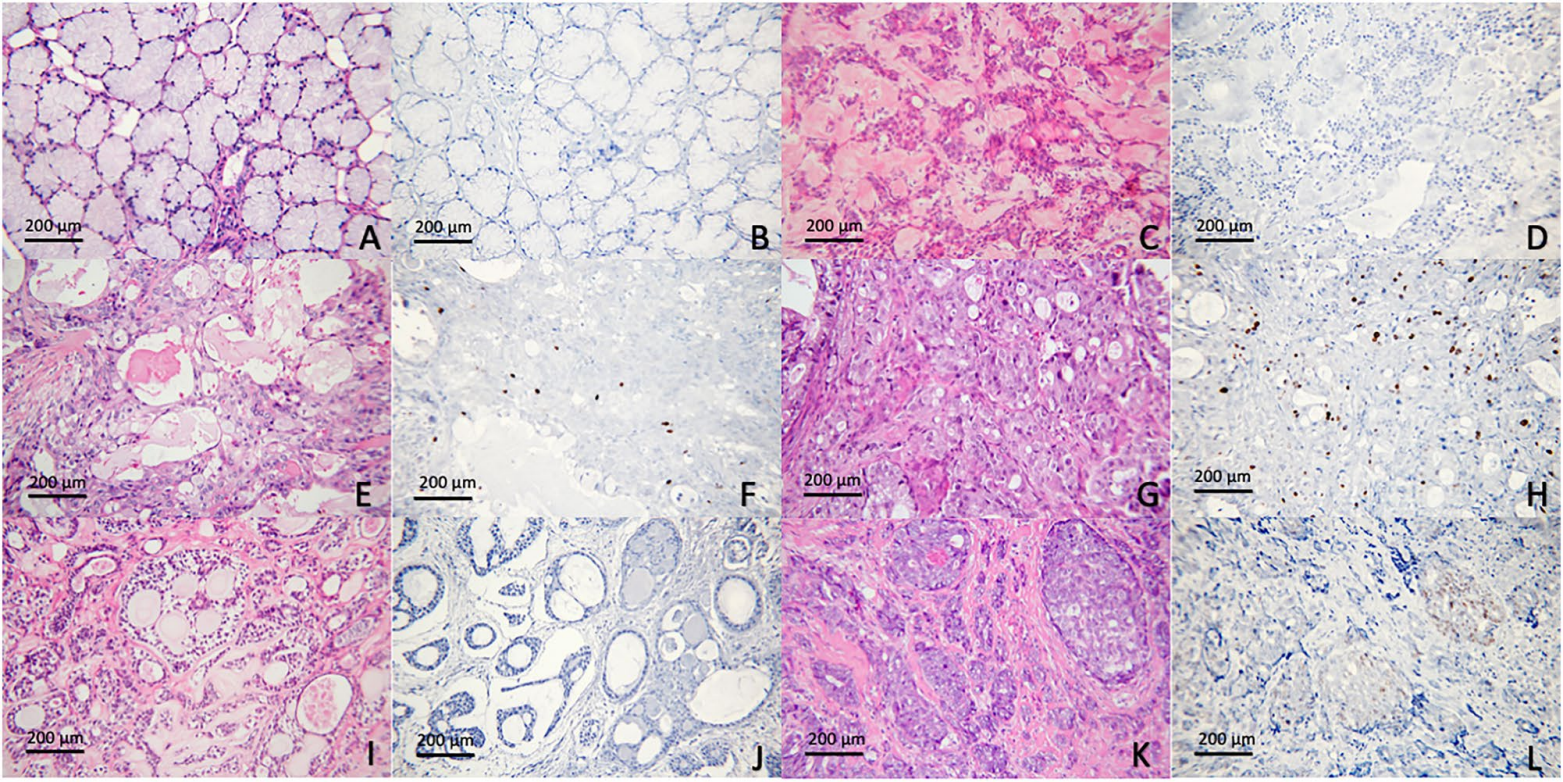
Ki-67 expression in salivary gland neoplasms. (**A**,**B**) Normal salivary gland tissue; (**C**,**D**) PA; (**E**,**F**) low-grade MEC; (**G**,**H**) high-grade MEC; (**I**,**J**) ACC, cribriform/tubular subtype; (**K**,**L**) ACC, solid subtype.

Ki-67 expression was determined to assess the proliferative activity of tumor cells. As expected, PA exhibited low proliferative activity, with Ki-67 LI ranging from 0–1.4 (mean = 0.11). In contrast, MEC and ACC showed higher Ki-67 LI, ranging from 0 to 25.38 (mean = 1.66) and from 0 to 17.9 (mean = 2.33), respectively. Notably, the intermediate-to-high grade MECs displayed greater proliferative activity (mean LI = 2.66) than low-grade cases (mean LI = 0.52). In addition, the solid-subtyped ACCs demonstrated increased proliferative activity (mean LI = 7.31), compared with ACCs with cribriform/tubular subtype (mean LI = 0.19). In normal salivary gland tissue, Ki-67 was not detectable (Fig. 1).

### Levels of histone H3 modifications in salivary gland neoplasms

**Figure 2:**
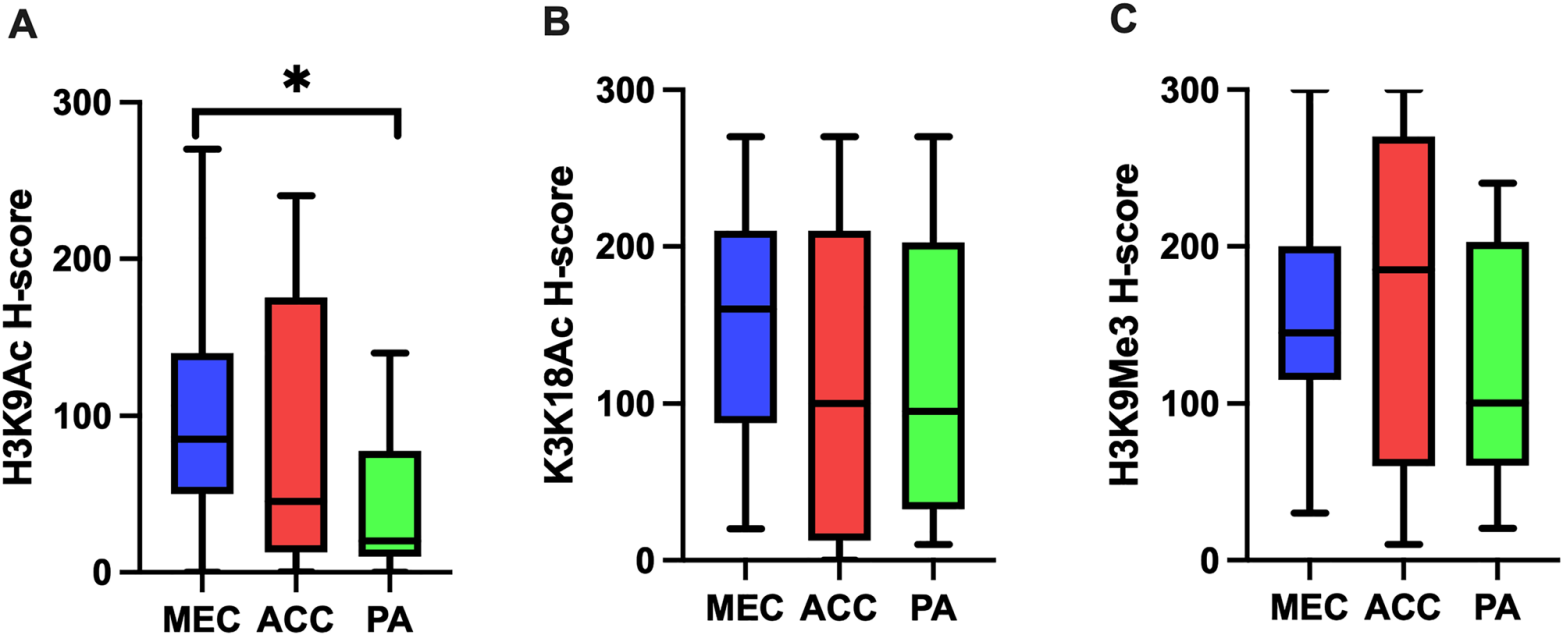
Level of histone H3 modifications in salivary gland neoplasms. (**A**) H3K9Ac; (**B**) H3K18Ac; (**C**) H3K9Me3. *Represents statistically significant difference at *p* < 0.05.

**Figure 3:**
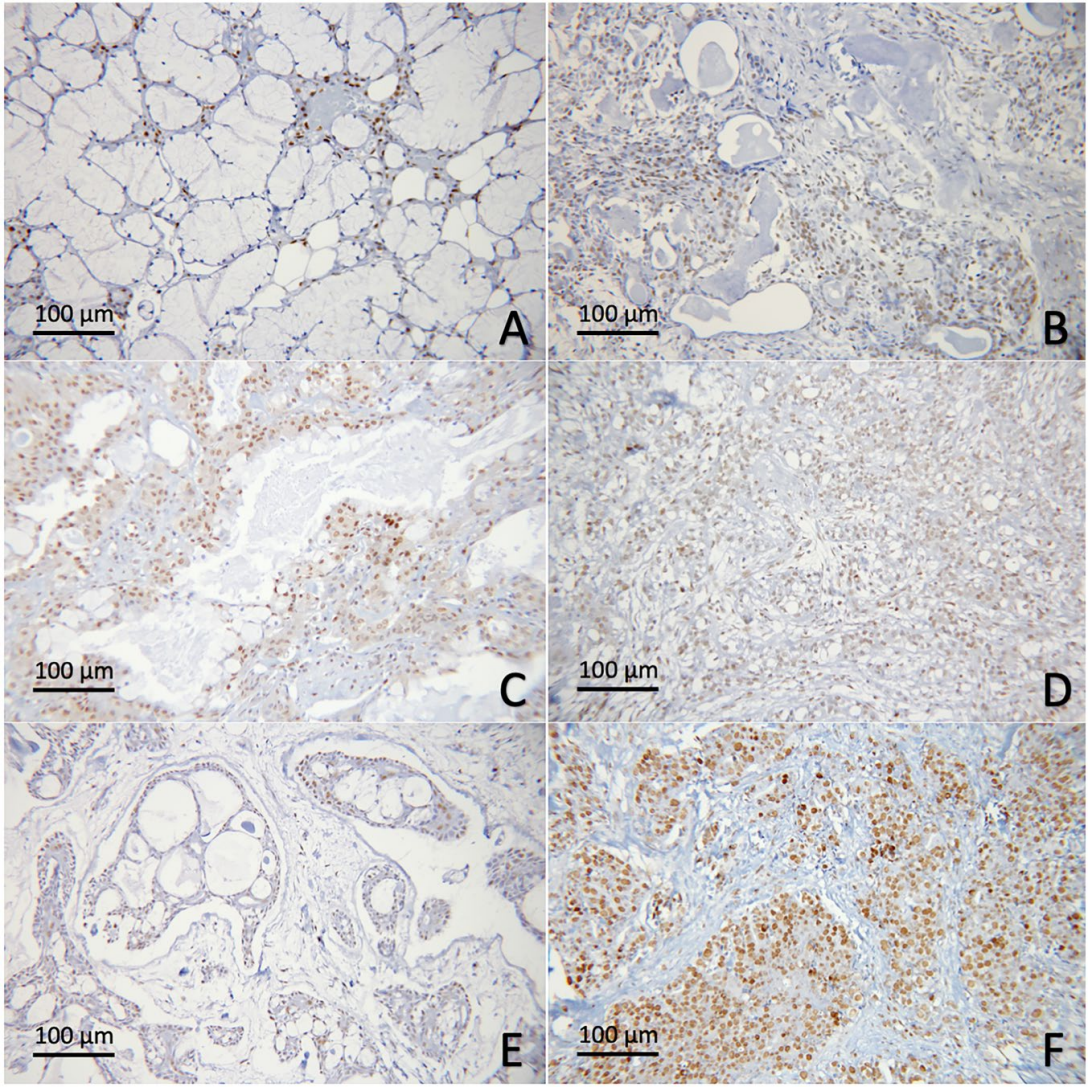
H3K9Ac in salivary gland neoplasms. (**A**) Normal salivary gland tissue; (**B**) PA; (**C**) low-grade MEC; (**D**) high-grade MEC; (**E**) ACC, cribriform/tubular subtype; (**F**) ACC, solid subtype.

Overall, we found nuclear expression of H3K9Ac in the majority of salivary gland neoplasms studied, constituting 93.3% of MECs, 85% of ACCs and 80% of PAs. The mean H3K9Ac H-scores for MEC, ACC and PA were 102.3 ± 71.2, 89.0 ± 87.4 and 39.5 ± 43.7, respectively. When compared the H3K9Ac expression between malignant and benign neoplasms, the malignant neoplasms demonstrated significantly higher H3K9Ac level (*p* = 0.003). Remarkably, H3K9Ac scores of MEC were significantly higher than those of PA (*p* = 0.004) (Figs. 2, 3).

**Figure 4:**
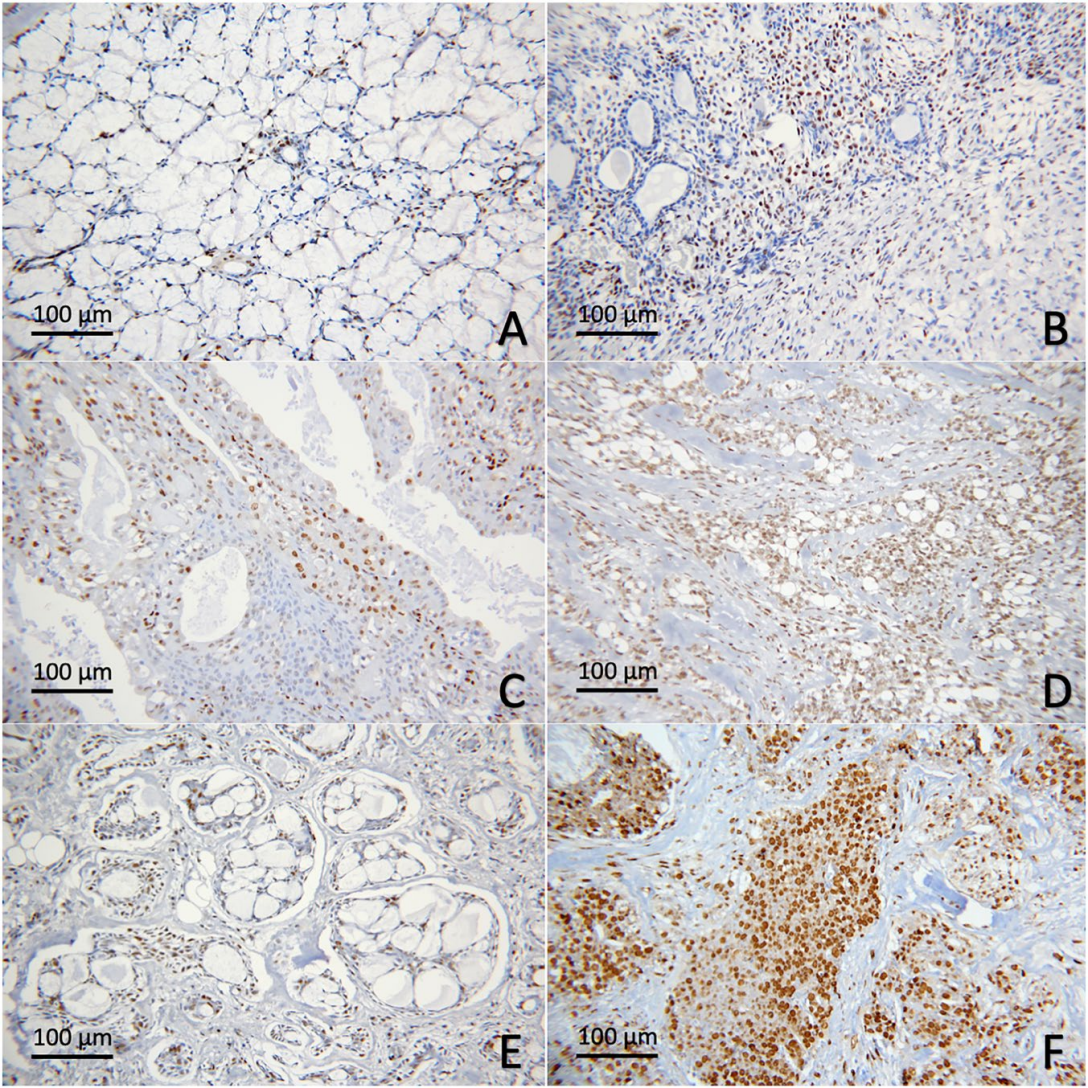
H3K18Ac in salivary gland neoplasms. (**A**) Normal salivary gland tissue; (**B**) PA; (**C**) low-grade MEC; (**D**) high-grade MEC; (**E**) ACC, cribriform/tubular subtype; (**F**) ACC, solid subtype.

H3K18Ac nuclear expression could be detected in neoplastic cells in all specimens, except one case of ACC. The mean H3K18Ac H-scores for MEC, ACC and PA were 147.7 ± 75.0, 115.5 ± 98.3 and 114.0 ± 87.8, respectively. No statistically significant differences were noted among the three neoplasms and between malignant and benign groups (Figs. 2, 4).

**Figure 5:**
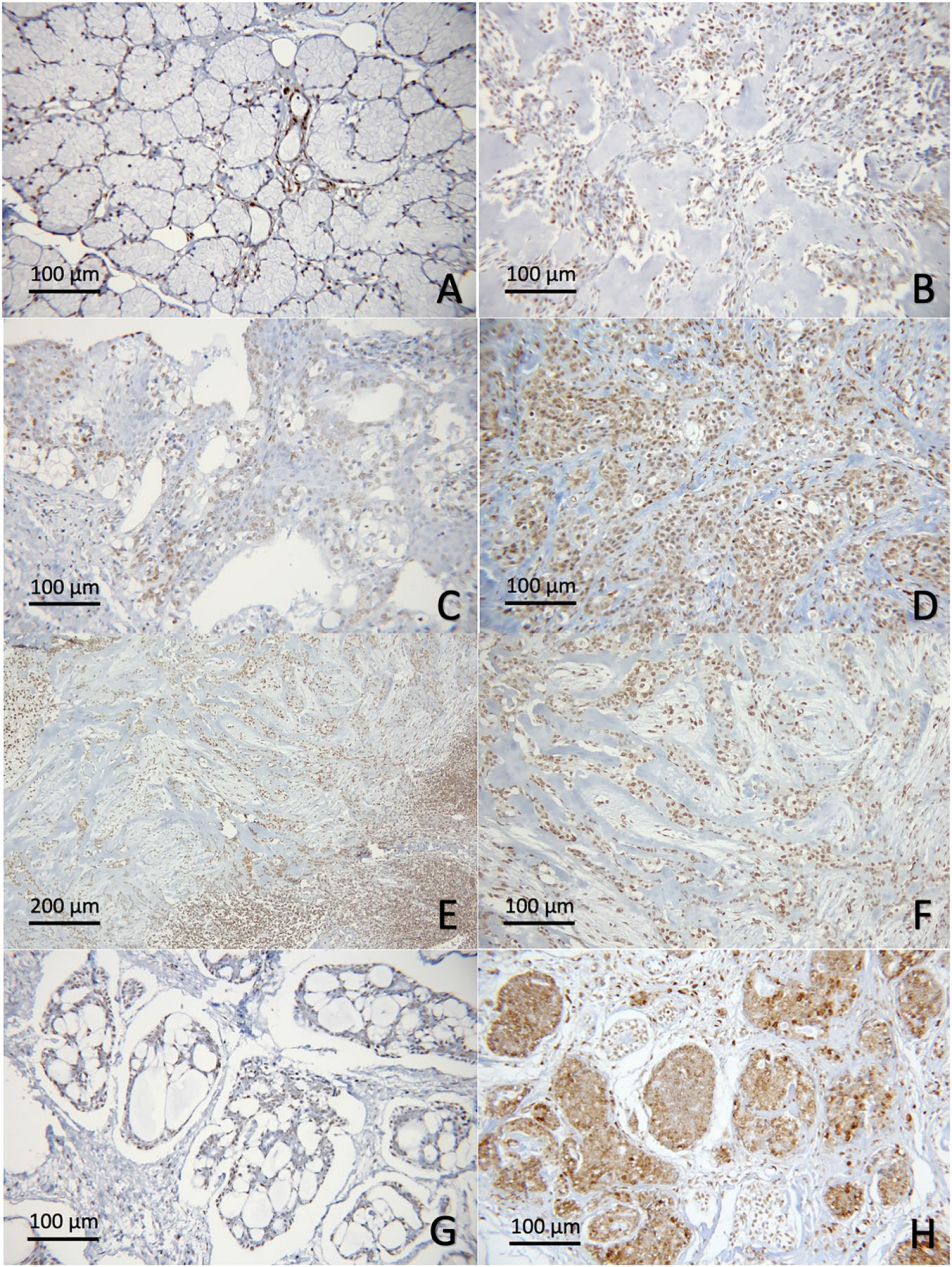
H3K9Me3 in salivary gland neoplasms. (**A**) Normal salivary gland tissue; (**B**) PA; (**C**) low-grade MEC; (**D**) high -grade MEC; (**E**,**F**) MEC with invasion into small nests at tumor fronts; (**G**) ACC, cribriform/tubular subtype; (**H**) ACC, solid subtype.

The nuclear expression of H3K9Me3 was observed in all specimens. The mean H3K9Me3 H-scores for MEC, ACC and PA were 152.7 ± 68.8, 168.0 ± 105.8 and 123.5 ± 72.8 respectively. Although PA appeared to express lower level of H3K9Me3, no statistically significant differences were detected among the three neoplasms and between malignant and benign neoplasms (Figs. 2, 5).

The cellular distribution for all types of histone modifications was investigated in both neoplasms and adjacent normal salivary gland tissues. Normal ductal and myoepithelial cells showed positive nuclear expression of all 3 markers tested. Positive stainings were also consistently detected in serous acinar cells, while normal mucous acini were largely non-reactive. In ACC and PA cases, histone modifications were variably seen in both neoplastic ductal and myoepithelial cells. In MEC, the positive immunoreactivity was relatively diverse among cases. These histone H3 modifications were noted primarily in the epidermoid and intermediate cells and variably expressed in neoplastic mucous cells (Figs. 3, 4, 5).

### The comparison between histone H3 modifications and histopathologic characteristics of malignant salivary gland neoplasms

Distinct histopathologic features of malignant salivary gland neoplasms can infer differences in tumor behavior. Therefore, we investigated the correlation between histone modification profiles and prognostically relevant histopathologic features of MEC and ACC. The results are summarized in Table 2. Significantly, we noted the upregulation of H3K9Me3 in MEC cases showing small nest invasion at tumor front (*p* = 0.017), the pattern indicative of more invasive cancer characteristics, and those with advanced pathologic grades (*p* = 0.028).

**Table 2:** The association between 3 types of histone H3 modifications and pathologic characteristics of MEC and ACC.

Pathologic characteristics	H3K9Ac		H3K18Ac		H3K9Me3	
	H-score	*P*-value	H-score	*P*-value	H-score	*P*-value
**MEC (N = 30)**	**102.3 ± 71.2**		**147.7 ± 75**		**152.7 ± 68.8**	
Pathologic grading						
Low (N = 14)	93.6 ± 49.1	1	130.7 ± 67.5	0.257	125.7 ± 53.5	***0.017***
Intermediate/high (N = 16)	110.0 ± 87.0		162.5 ± 80.1		176.3 ± 73.5	
< 25% cystic component						
Yes (N = 9)	80.0 ± 56.3	0.476	154.4 ± 79.5	0.722	153.3 ± 65.0	0.372
No (N = 21)	111.9 ± 75.9		144.8 ± 74.8		152.4 ± 71.9	
Tumor front invasion into small nests						
Yes (N = 16)	115.0 ± 85.087	0.552	163.1 ± 79.5	0.224	175.0 ± 74.3	***0.028***
No (N = 14)	0.9 ± 50.4		130.0 ± 68.0		127.1 ± 53.6	
Anaplasia						
Yes (N = 7)	65.7 ± 55.0	0.158	127.1 ± 81.4	0.501	157.1 ± 61.8	0.413
No (N = 23)	113.5 ± 72.8		153.9 ± 73.7		151.3 ± 72.0	
> 4 Mitosis per 10 HPF						
Yes (N = 1)	120	0.733	180	0.8	210	0.333
No (N = 29)	101.7 ± 72.4		146.6 ± 76.1		150.7 ± 69.1	
Vascular invasion						
Yes (N = 2)	60.0 ± 84.9	0.46	100.0 ± 113.1	0.414	120.0 ± 127.3	0.777
No (N = 28)	105.4 ± 70.9		151.1 ± 73.4		155.0 ± 66.3	
Perineural invasion						
Yes (N = 0)	–	N/A	–	N/A	–	N/A
No (N = 30)	102.3 ± 71.2		147.7 ± 75.0		152.7 ± 68.8	
Necrosis						
Yes (N = 2)	70.0 ± 99.0	0.662	115.0 ± 134.4	0.662	115.0 ± 120.2	0.662
No (N = 28)	104.6 ± 70.7		150.0 ± 72.7		155.4 ± 66.6	
**ACC (N = 20)**	**89.0 ± 87.4**		**115.5 ± 98.3**		**168.0 ± 105.8**	
Histopathologic subtypes						
Solid (N = 6)	160.0 ± 75.158	***0.012***	220.0 ± 31.0	***0.002***	250.0 ± 36.3	***0.041***
Cribriform/tubular (N = 14)	0.6 ± 75.2		70.7 ± 81.0		132.9 ± 106.9	
**PA (20)**	**39.5 ± 43.7**		**114.0 ± 87.8**		**123.5 ± 72.8**	

Significant values are in [bolditalics].

Remarkably in ACC, we found the statistically significant increase in all 3 types of histone H3 modifications, the H3K9Ac (*p* = 0.012), H3K9Me3 (*p* = 0.041) and H3K18Ac (*p* = 0.002), in the highly aggressive solid subtype, compared with the cribriform/tubular subtypes.

### The correlation between histone H3 modifications and Ki-67 in salivary gland neoplasms

**Figure 6:**
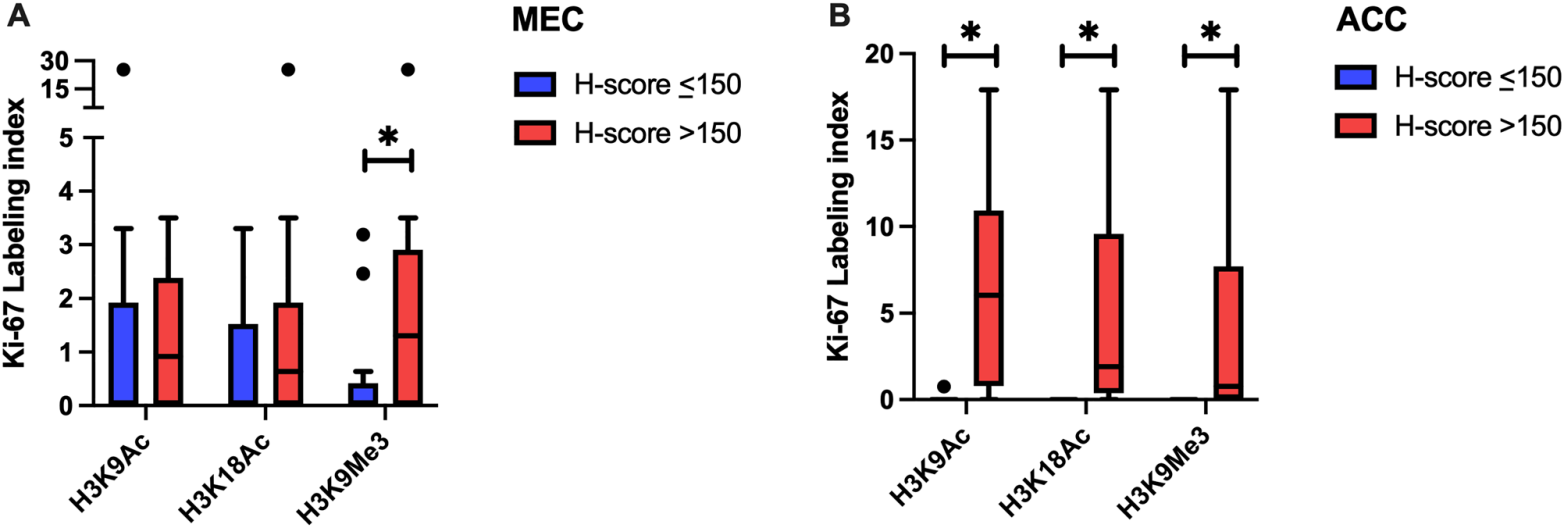
Ki-67 proliferative index of salivary gland malignancies according to the level of histone H3 modification. (**A**) MEC; (**B**) ACC. *Represents statistically significant difference at *p* < 0.05.

We investigated the relationships between the differential histone H3 modifications and the proliferative activity of salivary gland malignancies. The levels of histone modification were categorized into high (H-score >150) and low (H-score ≤150) groups. Remarkably, both the MEC and ACC with high level of histone H3 modifications demonstrated higher Ki-67 LI than those with low expression level. The differences reached statistically significant levels for H3K9Me3 in MEC and for all three histone H3 modifications in ACC (Figure 6). These data indicated that MECs with upregulated H3K9Me3 and ACCs with increased H3K9Ac, H3K18Ac and H3K9Me3 demonstrated higher tumor proliferative activity. The correlation between these histone H3 modifications and Ki-67 proliferative index was in concordance with the findings regarding their associations with aggressive pathologic characteristics described above.

### Predictive values of differential histone H3 modification in malignant salivary gland neoplasms

**Figure 7:**
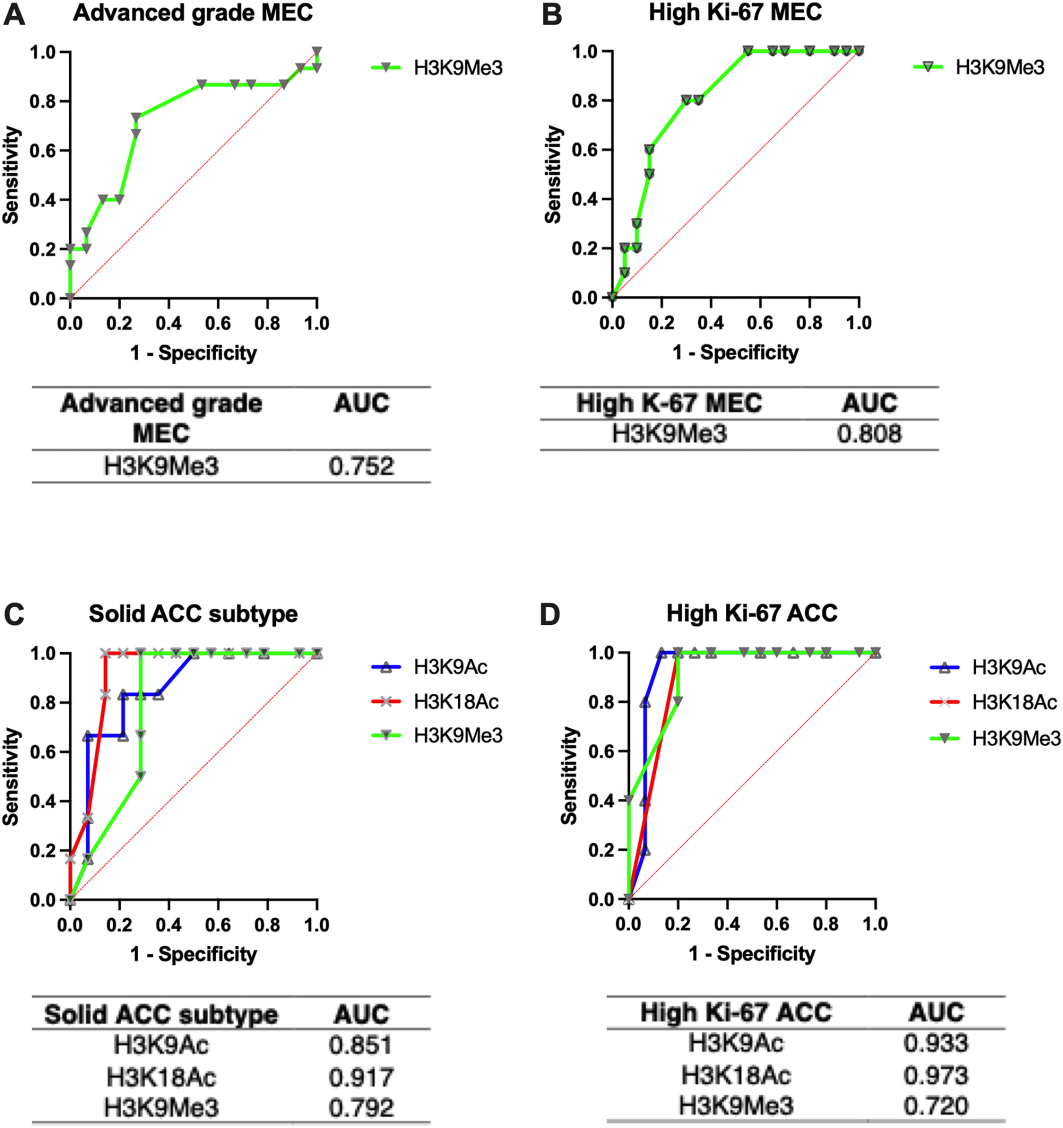
Receiver operating characteristic (ROC) curves and area under curves (AUC). The predictive assessments of the H3K9Me3 level and advanced pathologic grade MEC (**A**), the H3K9Me3 level and MEC with high Ki-67 proliferative index (**B**), the levels of 3 histone H3 modification and solid subtype of ACC (**C**), and the levels of 3 histone H3 modification and ACC with high Ki-67 proliferative index (**D**).

Since the levels of H3K9Me3 in MEC and H3K9Ac, H3K18Ac, H3K9Me3 in ACC were significantly associated with the aggressive pathologic characteristics and increased proliferative activity, we further assessed their predictive values using ROC curves and corresponding AUC values (Figure 7). We found that H3K18Ac exhibited the highest AUC value (0.917) in predicting solid type ACC, followed by H3K9Ac (AUC = 0.851) and H3K9Me3 (AUC = 0.792), respectively. Moreover, all three histone H3 modifications demonstrated excellent AUC values in predicting high Ki-67 ACC (0.933, 0.973 and 0.921 for H3K9Ac, H3K18Ac and H3K9Me3, respectively). In addition, H3K9Me3 demonstrated good (0.808) and fair (0.752) AUC values in predicting high Ki-67 MEC and high-grade MEC, respectively. These data suggested that H3K9Ac, H3K18Ac and H3K9Me3 could be promising prognostic indicators for the aggressive phenotypes of salivary gland malignancies.

### Correlation among 3 types of histone H3 modification in salivary gland neoplasms

The correlations among the histone H3 modification levels were analyzed in each tumor. The levels of H3K9Ac, H3K18Ac and H3K9Me3 showed significant correlation within the same tumor types as shown in Table 3. Interestingly, in ACC the strong correlations were observed among all 3 histone H3 modification types tested. In addition, the levels between H3K9Ac and H3K9Me3 and between H3K9Ac and H3K18Ac were highly correlated in MEC and PA, respectively.

**Table 3:** Correlation among 3 types of histone H3 modification in salivary gland neoplasms.

Histone modification	MEC		ACC		PA	
	R^2^	*P*-value	R^2^	*P*-value	R^2^	*P*-value
H3K9Ac-H3K9Me3	0.785	< 0.001	0.834	< 0.001	0.492	0.028
H3K9Me3-H3K18Ac	0.561	0.001	0.904	< 0.001	0.656	0.022
H3K9Ac-H3K18Ac	0.568	0.001	0.780	< 0.001	0.743	< 0.001

## Discussion

To our knowledge, this is the first study to simultaneously examine 3 distinct modifications of histone H3, the H3K9Ac, H3K18Ac and H3K9Me3, in the most common benign and malignant salivary gland tumors. Significantly, we demonstrate the novel findings that the upregulated H3K9Me3 is associated with the pathologically aggressive phenotypes and increased proliferative activity of both MEC and ACC. Overexpression of H3K9Me3 is detected in MEC cases showing invasive growth pattern as well as advanced pathologic grade, and in ACCs with the highly aggressive solid subtype. In addition to the H3K9Me3 upregulation, the solid-subtyped ACCs are also hyperacetylated at both H3K9 and H3K18, and these histone modifications are positively correlated with the proliferative index of tumor cells. Our findings substantiate that from the previous study showing amplified H3K9Me3 level in ACC cases with solid histopathologic pattern, distant metastasis and poor patient survival^23^. Overall, our data further advocate that the increased H3 trimethylation at lysine residue 9, as well as H3 acetylation at lysine residue 9 and 18, could be involved in the progression of these malignancies.

Methylation of histone often leads to the repressive mark, which suppresses gene expression^29^. H3K9Me3 is known to be associated with the heterochromatin and transcriptional silencing. During tumorigenesis, this modification could therefore regulate oncogenes or tumor suppressor genes and dictate their functions^30^. The prognostic impact of H3K9Me3 has been noted in other cancers, such as gastric adenocarcinoma^10^, pancreatic cancer^31^ and esophageal squamous cell carcinoma^32^. Selected mechanisms were proposed for the oncogenic role of H3K9Me3. Overexpression of H3K9Me3 and/or its methyltransferases, SETDB1 or SUV39H1/2, was shown to silence the expression of tumor suppressor genes, including p53^33^, HoxA^34^, Kruppel-like transcription factor 6 (KLF6)^35^ and p15INK4B and E-cadherin^36^ in liver cancer cells, melanoma, liposarcoma and acute myeloid leukemia, respectively. Paschall et al*.* reported that the upregulated H3K9Me3 suppressed Fas expression in metastatic colon cancers and involved in cancer chemoresistance^37^. A study on verticillin A, a selective inhibitor of SUV39H1, reported its effect on the increased FAS transcription and apoptosis of colon carcinoma cells^37^. However, it has not yet been tested in human or in the context of salivary gland tumors. In addition, the downregulation of SETDB1 could impede cancer cell proliferation in those of lung^38^, breast^39^ and prostate gland^40^. A previous study in lung cancer also observed the association between the increased methylation of H3K9 and epithelial cell adhesion molecule (EpCAM) silencing, the event of which promoted cancer invasion and metastasis^41^. Notably, EpCAM was shown to be downregulated in high-grade MEC showing aggressive pathologic characteristics^42^. Moreover, in salivary gland malignancies, the reduced expression of several tumor suppressor genes such as CDKN2A/p16, APC, Mint1, PGP9, RAR-β, Timp3 have been reported^43^. In conjunction with our data, it should be of interest in future studies to investigate the functional involvement of hypertrimethylated H3K9 on EpCAM and other tumor suppressor genes in relation to progression of salivary gland cancers. Additionally, the expression of SUV39H and SETDB1 in salivary gland malignancies should be of value for potential application of targeted treatment in patients with these cancers.

In the present study, we also note the significantly greater H3K9Ac level in MEC than benign PA, and that the solid-typed ACCs upregulated both H3K9Ac and H3K18Ac. Previous studies demonstrated variable roles of histone acetylation in carcinogenesis. Acetylation of histone leads to the open chromatin and increased gene expression^44^. Its effects can be varied, depending on the acetylation sites as well as the tumor types. While the association between diminished H3K9Ac and poor prognosis had been observed in selected malignancies, such as those of breast^4^ and oral mucosa^45^, studies in gastric adenocarcinoma^10^, grade I lung adenocarcinoma^2^ and colorectal cancer^46^ reported findings corresponding to ours, that the hyperacetylated H3K9 inferred worse cancer outcomes. Regarding H3K18Ac, its overexpression was associated with poor prognostic factors in several cancers, including thyroid cancer^47^, prostate cancer^3^, glioma^48^, hepatocellular carcinoma^49^ and oral squamous cell carcinoma^50^. Nevertheless, other types of cancer, including breast cancer^4^ and colorectal cancer^51^ demonstrated a reverse relationship, and varying results among studies were observed in pancreatic cancer^6,8^.

In addition to the H3K9Me3, our findings supported the potential role of H3K9Ac and H3K18Ac in solid-typed ACC formation and the significantly increased tumor cell proliferation. Hyperacetylation at these histone sites could activate transcription of certain oncogenic pathways. Interestingly, a study in liver cancer model showed that tumor cells that failed at H3K9Ac/H3K9Me3 transition, could lead to the hyperacetylation of H3K9 and increased expression of many oncogenes such as Kras, Ercc1, Cdk6, Usp39, and Mapre3^52^. In addition, previous studies reported that levels of H3K9Ac and H3K18Ac were correlated with the increased transcription of genes, which could be involved in salivary gland carcinogenesis, such as NOTCH1 (both H3K9Ac and H3K18Ac)^53,54^, MUC1 (H3K9Ac)^55^, c-MYB (H3K9Ac)^56^ and EGFR downstream protein (H3K18Ac)^54,57–59^. Moreover, the MECT1-MAML2, fusion oncogene commonly detected in MEC^60^, was shown to recruit and activate activity of CBP/p300, which is an H3K18 acetyltransferase^61,62^. However, the exact mechanisms are not yet known. These data could establish the foundation for future research on the roles these histone modifications may play in ACC progression.

Histone H3 acetylation in salivary gland tumors had been investigated by a handful of studies with variable results. Corresponding to our findings, Kishi et al*.* demonstrated that global H3 acetylation was frequent in MEC (71.4%) and ACC (76.5%)^63^. However, Xia et al*.* reported that low H3K9Ac expression in ACC was correlated with poor prognosis^23^. These variable findings could be resulted from the differences in tissue source and immunohistochemical evaluation scheme. In this study, we used whole tissue specimens to quantify the levels of histone modification, based on both the tissue distribution and staining intensity. While examining these tissue sections, we noticed discernable variation in the distribution and intensity of immunoreactivity within the same sections. This could be due to the inherent cellular heterogeneity of salivary gland neoplasms, since each tumor is distinctly composed of the admixture of several cell types with different morphologic features and contrasting levels of histone modification from one area to another. H3K9Ac expression in particular was shown to be heterogeneous in tissue specimens, especially those from high-grade tumors^46^. This heterogenous pattern of expression could well be affected by sampling (whole tissue sections vs. tissue microarray) and immunohistochemical staining scoring methods^64^.

Studies on the level of key enzymes responsible for these modified histones are limited. SETDB1 gene expression was shown to be higher in PA tissues, compared with normal salivary glands^24^. A recent study on HDACs reported the association of HDAC-2 with better overall survival of patients with salivary gland malignancies, whereas the increased HDAC-6 expression indicated poor prognosis^25^. However, the interpretation of these findings in relation to global histone modification data may not be straightforward. Both HDAC-2 and HDAC-6 were known to be able to deacetylate H3K9^65–67^, H3K18^68^, as well as other histone and non-histone proteins^69–71^. Due to their complex substrate specificity and largely undetermined recognition sites, investigating the correlation of among the expression of all HDACs and their substrates would be challenging and yet to be further investigated^71^.

Our data strongly support the connection that acetylation of histone H3 plays in salivary gland carcinogenesis and could shed some light on potential novel treatment. There have been clinical trials using HDAC inhibitor in treating ACC and MEC with limited success^72,73^. Vorinostat, an HDAC inhibitor, efficiently disrupted the population of cancer stem cells, but did not significantly reduce the total number of tumor cells^74^. Our findings suggest that there is a level of epigenetic heterogeneity within these cancers, and it could also be beneficial to investigate the use of Sirtuins (SIRT1-7), a class of deacetylases selectively targeting H3K18, as another potential therapeutic option at least in selected cases^75^. Results from previous studies together with our study signify that both MEC and ACC govern diverse genetic and epigenetic alterations and are inherently heterogenous with lesions. Moreover, the regulation of gene expression is complex and there are other types of epigenetic controls possibly into play, such as DNA methylation, miRNAs, etc^76^. Individual tumors may acquire different genetic and epigenetic signatures. Our findings emphasize the need to screen for all potential genetic and epigenetic alterations in each individual before choosing the targeted treatment for salivary gland carcinomas.

Our study carried some limitations. We examined global histone modifications without detection of specific gene alterations. However, immunohistochemistry allowed us to assess the expression pattern and cellular localization of these histone modifications throughout the entire tumor specimens. Although no long-term patient follow-up could be obtained, the prognostically relevant histopathologic features and proliferative activity were used to correlate with levels of histone modification.

In conclusion, our results support that epigenetic alteration is crucial for the regulation of salivary gland tumorigenesis. Hyperacetylation and trimethylation of histone H3 are associated with the increased tumor proliferative ability and the aggressive solid-subtyped formation in ACC and could be used as potential prognostic markers or future targeted therapy of this neoplasm. Notably, H3K9Me3 could be involved in the increased cancer proliferation, invasive characteristics, and advanced grade of MEC.

## Data Availability

The datasets used and analyzed during the current study available from the corresponding author on reasonable request.
